# Path tracking control method for tracked agricultural vehicles based on slip-aware look-ahead point offset

**DOI:** 10.3389/fpls.2025.1754679

**Published:** 2026-01-05

**Authors:** Huanyu Liu, Zhihang Han, Jiaqin Yin, Junwei Bao, Jian Mu, Hewen Tan, Xiangnan Liu

**Affiliations:** 1 Institute of Modern Agricultural Equipment, Xihua University, Chengdu, China; 2 Inner Mongolia Academy of Agricultural and Animal Husbandry Sciences, Hohhot, China

**Keywords:** extended Kalman filter, improved particle swarm optimization, pure pursuit algorithm, slip compensation, tracked agricultural machinery

## Abstract

**Introduction:**

Tracked agricultural vehicles operating in complex farmland environments are prone to track slip, which degrades path-tracking accuracy and may lead to unstable motion. To address the limitations of conventional geometric tracking algorithms under slip conditions, this study proposes a slip-aware look-ahead point offset path-tracking control method for tracked agricultural machinery.

**Methods:**

An extended Kalman filter (EKF) is developed to fuse RTK–IMU pose measurements with track wheel-speed feedback, enabling real-time estimation of left and right track slip ratios. Based on the estimated slip difference, a target-point offset compensation mechanism is constructed, and the offset angle is optimized online using an improved particle swarm optimization (PSO) algorithm with a Chebyshev-window-based inertia weight strategy. In addition, a fuzzy controller is employed to adaptively adjust the look-ahead distance according to vehicle speed and path curvature, while a first-order low-pass filter is applied to smooth the commanded velocities.

**Results:**

Simulation results demonstrate that the proposed method significantly reduces lateral tracking errors and maintains smooth trajectories under severe slip conditions. Field experiments conducted at speeds of 0.35 m/s and 0.75 m/s show that the proposed method reduces the maximum lateral deviation by 78.1% and the average deviation by 50.6% compared with the traditional fuzzy pure pursuit algorithm. At 0.75 m/s, the maximum and average deviations are further reduced by 63.1% and 57.6%, respectively.

**Discussion:**

The results confirm that incorporating slip estimation and slip-aware target-point offset compensation effectively enhances path-tracking accuracy and robustness for tracked agricultural vehicles operating on soft and high-slip terrain. The proposed lightweight control framework provides a practical and reliable solution for autonomous navigation and plant-protection operations in complex farmland environments.

## 1 Introduction

With the advancement of agricultural modernization and intelligence, the issues of autonomous navigation and path tracking for tracked agricultural machinery in complex farmland environments have attracted increasing attention (Sun et al., 2024). As a critical component of unmanned agricultural operations, path tracking control directly influences both the operational quality and overall efficiency (Zhang et al., 2020; Shi et al., 2023; Chen and Wang, 2024). However, due to the soft terrain and complex adhesion conditions typically encountered in farmland operations, tracked vehicles inevitably experience varying degrees of slip during motion. This slip causes deviations between the actual trajectory and the desired path and may even lead to control instability (Jiao et al., 2018; Zhao et al., 2024). Therefore, achieving high-accuracy and robust path tracking control under slip disturbances has become an urgent research challenge.

A substantial amount of research has been conducted on path tracking control for agricultural machinery both domestically and internationally (Wang et al., 2019; Liu et al., 2023; Xu et al., 2023; Liu et al., 2025b). Classical approaches such as the Pure Pursuit and Stanley algorithms have been widely adopted due to their simple structure and good real-time performance. Zhang et al (Zhang et al., 2024) proposed an integral-compensation fuzzy Pure Pursuit method based on B-spline path optimization, where path interpolation and fuzzy adaptive adjustment of the look-ahead distance effectively reduced lateral deviation during curved-path tracking. Song et al (Song et al., 2025) employed the sparrow search algorithm to adaptively tune the look-ahead distance, achieving average lateral and heading errors of 0.03 m and 0.275°, respectively. Cui et al (Cui et al., 2022) developed an improved Stanley model incorporating fuzzy inference, in which the control gain is adjusted in real time based on lateral and heading errors, thereby enhancing curve-segment tracking accuracy and adaptability in full-field agricultural operations. Wang et al (Wang et al., 2022) proposed an improved Stanley controller (IMP-ST) and optimized its parameters using a multi-population genetic algorithm (MPGA), resulting in a 48.61% improvement in tracking performance. In addition to control-oriented improvements, researchers have also explored robust path planning frameworks for agricultural robots. Yang et al. proposed AgriPath, a multi-objective path planning framework designed for dynamic field environments, which integrates multimodal perception and optimization techniques to enhance the adaptability and safety of agricultural robot navigation (Yang et al., 2025).

However, such methods generally assume no slip, leading to a significant decline in tracking accuracy under actual farmland conditions. Path tracking approaches based on model predictive control (MPC) can account for vehicle dynamics and operational constraints to some extent, but their high computational complexity makes it difficult to meet the real-time requirements of agricultural operations (Bai et al., 2020). He et al (He et al., 2022) developed an MPC-based path controller using attitude measurements in paddy fields, achieving an average absolute error of 0.033 m in field experiments. In recent years, MPC has also attracted increasing attention in autonomous driving and mobile robot navigation due to its capability of handling multivariable constraints and nonlinear dynamics.

Recent studies have shown that MPC-based frameworks can achieve outstanding tracking precision and strong robustness even under high-dynamic or constrained operating conditions. For instance, Guo et al (Guo et al., 2025) demonstrated that a Lyapunov-based nonlinear MPC formulation can provide highly stable and accurate trajectory tracking for distributed-drive electric vehicles, effectively ensuring handling performance under complex dynamic interactions. Similarly, Wang and Xiong developed a coupled MPC controller for four-wheel-steering vehicles and verified its capability in coordinating multi-degree-of-freedom motions while maintaining high tracking accuracy in dynamic scenarios. These works collectively highlight the advantages of MPC in constrained planning and control. Nevertheless, despite these strengths, the computational burden of MPC remains a major obstacle for real-time deployment on lightweight agricultural platforms.

In addition, some researchers have attempted to improve control accuracy through slip modeling and compensation (Jiao et al., 2015), such as incorporating Kalman filtering (Qian et al., 2024)or applying fuzzy controllers for adaptive adjustment (Zhang et al., 2021). Li et al (Li et al., 2023) proposed an adaptive temporal regulation strategy for orchard scenarios, achieving tracking errors within 5 cm in field tests. Nevertheless, the compensation capability of existing methods remains limited under complex slip conditions.

To address the above issues, this study proposes a slip-aware look-ahead point offset path tracking control method for tracked agricultural machinery. In the method design, a fuzzy controller is first developed to adaptively adjust the look-ahead distance, accommodating the response characteristics of the Pure Pursuit algorithm under varying speeds and path curvatures. It is worth noting that fuzzy control has been widely applied in path tracking research (Li et al., 2013), and its introduction here serves as a commonly used baseline optimization rather than the core innovation of this work. Building on this, an extended Kalman filter integrating RTK–IMU measurements with track wheel-speed feedback is constructed to estimate the slip ratios of the left and right tracks in real time. Subsequently, a target-point offset compensation mechanism is innovatively proposed, which takes the differential slip ratio between the two tracks as the input, while an improved particle swarm optimization algorithm is employed to obtain the optimal offset angle, thereby enhancing control accuracy and robustness under slip conditions. In addition, a first-order low-pass filter is incorporated into the velocity control loop to improve the smoothness of the commanded signals. Simulation and field experiments are conducted to validate the path tracking accuracy and stability of the proposed algorithm in complex farmland environments.

The main contributions of this work are summarized as follows:

1. A slip-aware look-ahead point offset mechanism is proposed to compensate for trajectory deviation under asymmetric track slip. The offset angle is optimized by an improved PSO algorithm with a Chebyshev-window-based inertia weight strategy.
2. An extended Kalman filter integrating RTK–IMU pose data with track wheel-speed feedback is developed to estimate the left–right track slip ratios in real time, providing reliable slip-state perception for the controller.
3. A complete lightweight path-tracking control framework is constructed by combining adaptive look-ahead tuning, slip estimation, offset compensation, and velocity filtering. Both simulation and field experiments verify the accuracy, robustness, and engineering feasibility of the proposed method under complex farmland conditions.

The remainder of this paper is organized as follows. Section 2 introduces the system modeling and overall control framework. Section 3 presents the design of the key algorithms, including the adaptive look-ahead strategy, slip estimation, and offset optimization. Section 4 provides simulation and experimental validation. Section 5 concludes the paper and discusses future research directions.

## 2 Overall design of the path tracking system

To achieve stable path tracking control for tracked agricultural machinery in complex farmland environments, a complete control system architecture must be established, covering vehicle modeling, target-point computation, and controller design. This section focuses on the design of the path tracking system, beginning with the kinematic modeling of the tracked vehicle, followed by an introduction to the classical Pure Pursuit control method. An improved path tracking control framework incorporating slip-awareness is then presented to address the limitations of conventional approaches under slip conditions.

### 2.1 Kinematic model of the tracked agricultural vehicle

Before designing the path tracking control algorithm, it is necessary to establish the kinematic model of the tracked agricultural vehicle to characterize its pose evolution and provide a theoretical basis for controller development (Liu et al., 2025a). The tracked vehicle performs steering through differential driving of the left and right tracks, and based on its structural characteristics, it can be abstracted as a typical differential-drive model. For the convenience of modeling and analysis, the following reasonable assumptions are made for the tracked vehicle:

- The vehicle is regarded as a rigid-body system, and elastic deformation during motion is not considered; the center of mass is assumed to remain fixed at the midpoint of the chassis.
- The motion of the vehicle is constrained to a horizontal plane, where only planar translation and rotation about the vertical axis are considered.
- The vehicle adopts a differential steering mechanism, and turning is achieved by controlling the linear velocity difference between the left and right tracks; elastic coupling and asymmetric wear between the two tracks are not considered.
- In the basic model described in this section, lateral sideslip is assumed to be absent, and the velocity of the center of mass is aligned with the longitudinal axis of the vehicle. In subsequent sections, slip ratio parameters and estimation models will be introduced based on this fundamental model to better approximate real operating conditions.

**Figure 1:**
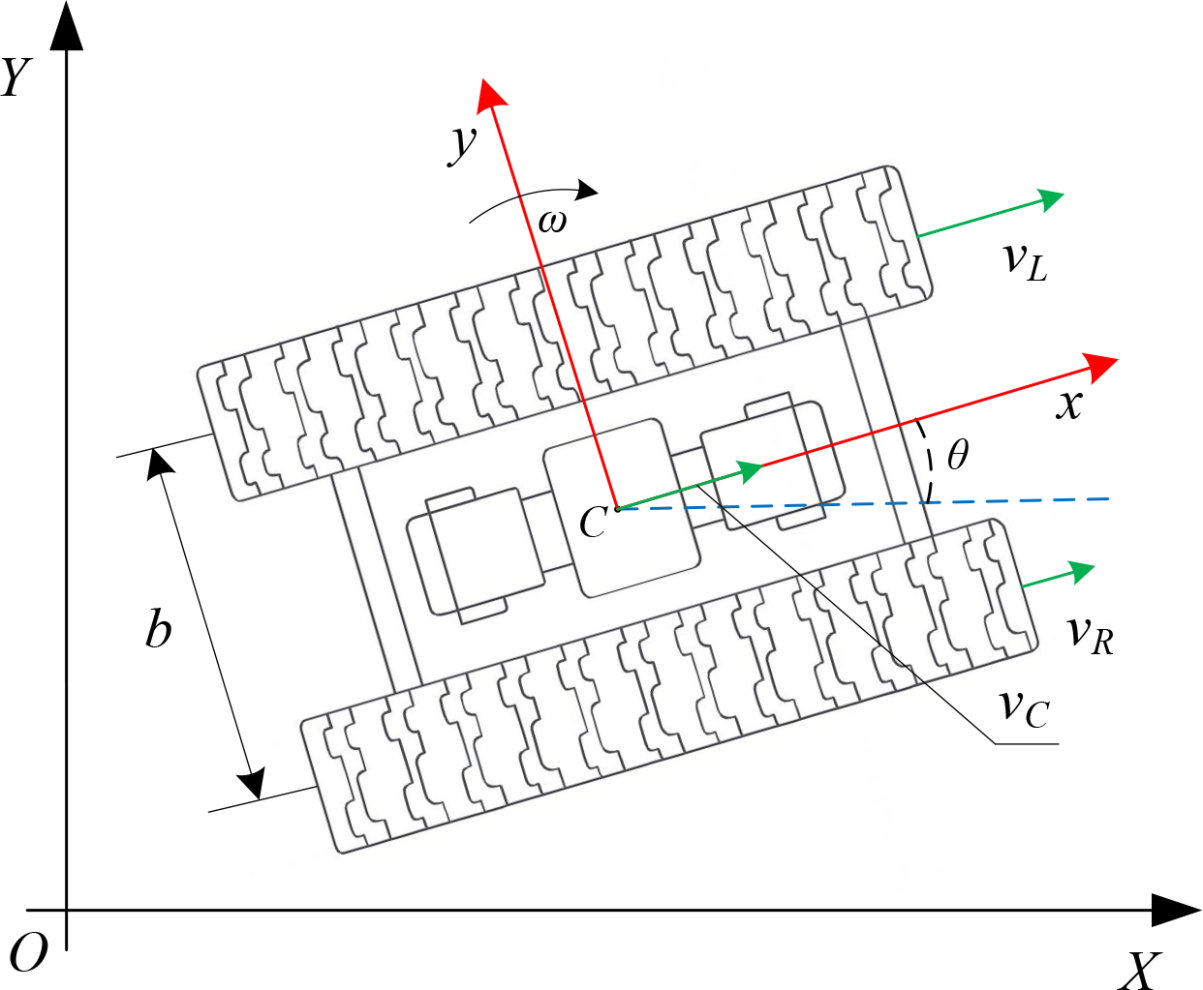
Kinematic modeling schematic of a tracked agricultural vehicle.

The kinematic schematic is illustrated in **Figure 1**, where the vehicle’s center of mass is defined as the reference point C. A body-fixed coordinate frame (x, y) is established, and the heading angle *θ* is defined as the angle between the body-frame *x*-axis and the ground-fixed *X*-axis. The linear velocities of the left and right tracks are denoted as *v_L_*"and *v_R_*, respectively. The forward velocity of the vehicle’s center of mass along the body-frame longitudinal axis is represented by *v_c_*, and the angular velocity of the vehicle is denoted as *ω*.

The velocity of the vehicle’s center of mass is obtained as the average of the left and right track velocities, while the angular velocity is generated by their velocity difference. The expressions are given as shown in Equation 1.

(1) $$\left\{ \begin{array}{l} v_{C}=\frac{v_{R}+v_{L}}{2}\\ \omega =\frac{v_{R}-v_{L}}{b} \end{array}\right.$$

Accordingly, the rate of change of the vehicle’s position in the global coordinate frame and the rate of change of its heading angle can be expressed as shown in Equation 2.

(2) $$\left\{ \begin{array}{l} \dot{x}=v_{C}\cos\theta =\frac{v_{R}+v_{L}}{2}\cos\theta \\ \dot{y}=v_{C}\sin\theta =\frac{v_{R}+v_{L}}{2}\sin\theta \\ \dot{\theta }=\omega =\frac{v_{R}-v_{L}}{b} \end{array}\right.$$

### 2.2 Pure pursuit tracking model

**Figure 2:**
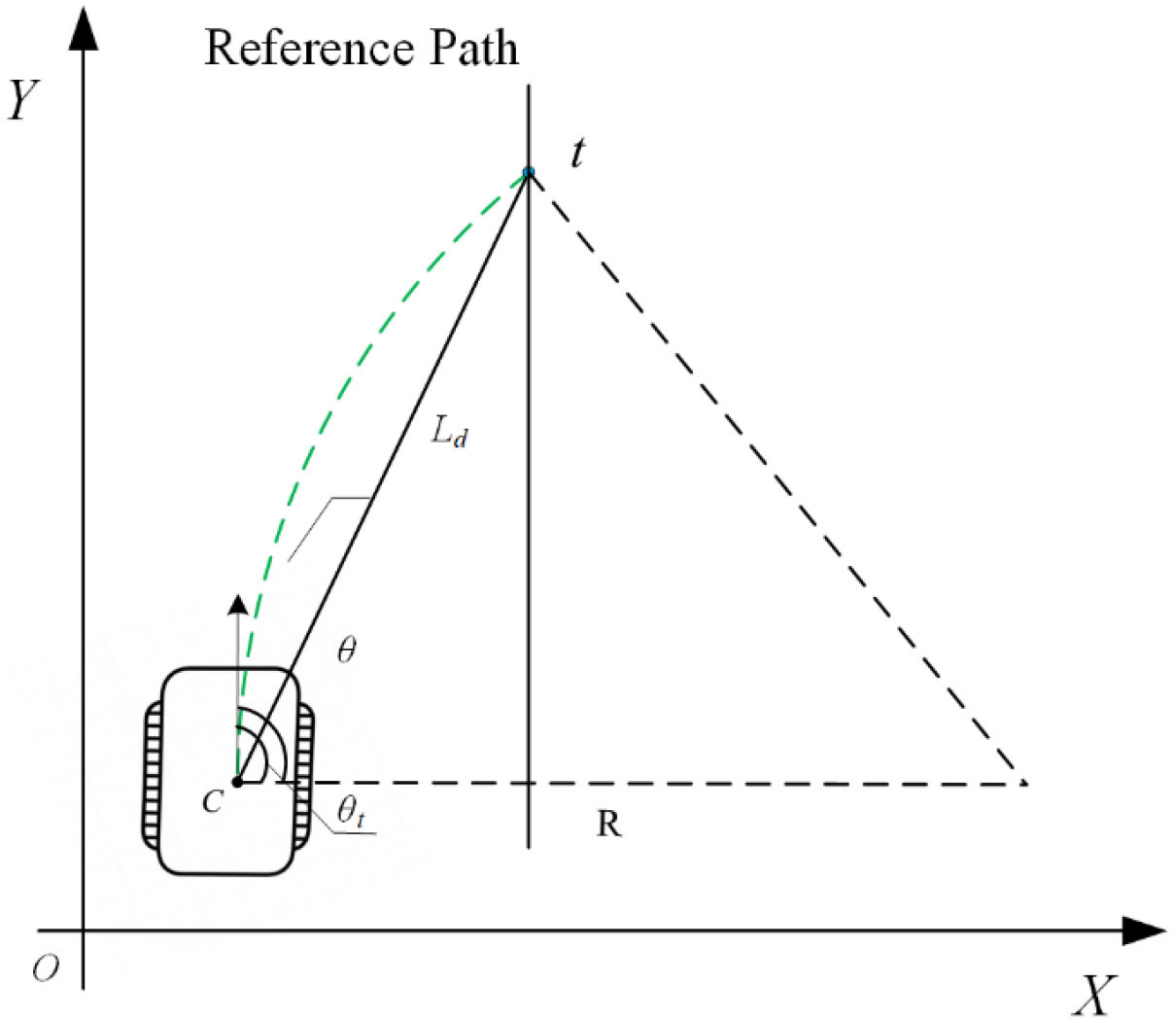
Geometric relationship schematic of the pure pursuit path-tracking algorithm.

The Pure Pursuit algorithm is a geometry-based path tracking method whose core idea is to steer the vehicle toward a target point located ahead on the reference path (Hu et al., 2024). For tracked agricultural machinery, the Pure Pursuit controller is combined in this study with the differential-drive kinematic model to derive the control formulas for the left and right track velocities. As illustrated in **Figure 2**:

Assuming that the current position of the vehicle’s center of mass is (*x_C_, y_C_*) with a heading angle *θ*, the current look-ahead distance is *L_d_*, and the target point position is (*x_t_, y_t_*), the direction from the vehicle to the target point can be expressed as shown in Equation 3.

(3) $$\theta_{t}=\arctan(y_{t}-y,x_{t}-x)$$

Accordingly, the yaw error between the vehicle and the target point is given as shown in Equation 4.

(4) $$\alpha =\theta_{t}-\theta$$

The vehicle turns with a radius R around the circumscribed circle passing through the target point, and the distance between the target point and the vehicle’s center of mass is the look-ahead distance *L_d_*. According to the geometric relationship, Equation 5 can be obtained.

(5) $$\sin\alpha =\frac{L_{d}}{2R}\Rightarrow R=\frac{L_{d}}{2\sin\alpha }$$

According to the definition of the turning radius for a tracked vehicle, the relationship between the angular velocity and the turning radius is given as shown in Equation 6.

(6) $$\omega =\frac{v_{c}}{R}$$

Substituting Equation 5 into the corresponding expression yields Equation 7.

(7) $$\omega =\frac{2v_{c}\sin\alpha }{L_{d}}$$

When the vehicle exhibits angular velocity, the two tracks rotate about the same instantaneous center of curvature (ICC) with respect to the vehicle’s center of mass. The rotational radii of the left and right tracks are given as shown in Equation 8.

(8) $$\left\{ \begin{array}{l} R_{L}=R-\frac{b}{2}\\ R_{R}=R+\frac{b}{2} \end{array}\right.$$

Accordingly, the velocities of the left and right tracks can be expressed as shown in Equation 9.

(9) $$\left\{ \begin{array}{l} v_{L}=\omega R_{L}=v_{c}-\omega \cdot \frac{b}{2}\\ v_{R}=\omega R_{R}=v_{c}+\omega \cdot \frac{b}{2} \end{array}\right.$$

Substituting Equation 7 into the corresponding expression yields Equation 10.

(10) $$\left\{ \begin{array}{l} v_{L}=v-\frac{b}{2}\cdot \frac{2v_{c}\sin\alpha }{L_{d}}=v_{c}(1-\frac{b\sin\alpha }{L_{d}})\\ v_{R}=v+\frac{b}{2}\cdot \frac{2v_{c}\sin\alpha }{L_{d}}=v_{c}(1+\frac{b\sin\alpha }{L_{d}}) \end{array}\right.$$

### 2.3 Structure of the slip-aware path tracking control system

The traditional Pure Pursuit algorithm can achieve satisfactory path tracking performance under the ideal assumption of no slip. However, in soft and complex farmland terrain, tracked vehicles inevitably experience varying degrees of slip on the left and right tracks, resulting in deviations between the actual and desired trajectories and potentially causing system divergence (Zhao et al., 2025). Therefore, based on the Pure Pursuit controller, this study proposes an improved path tracking control system that incorporates a slip-aware mechanism.

**Figure 3:**
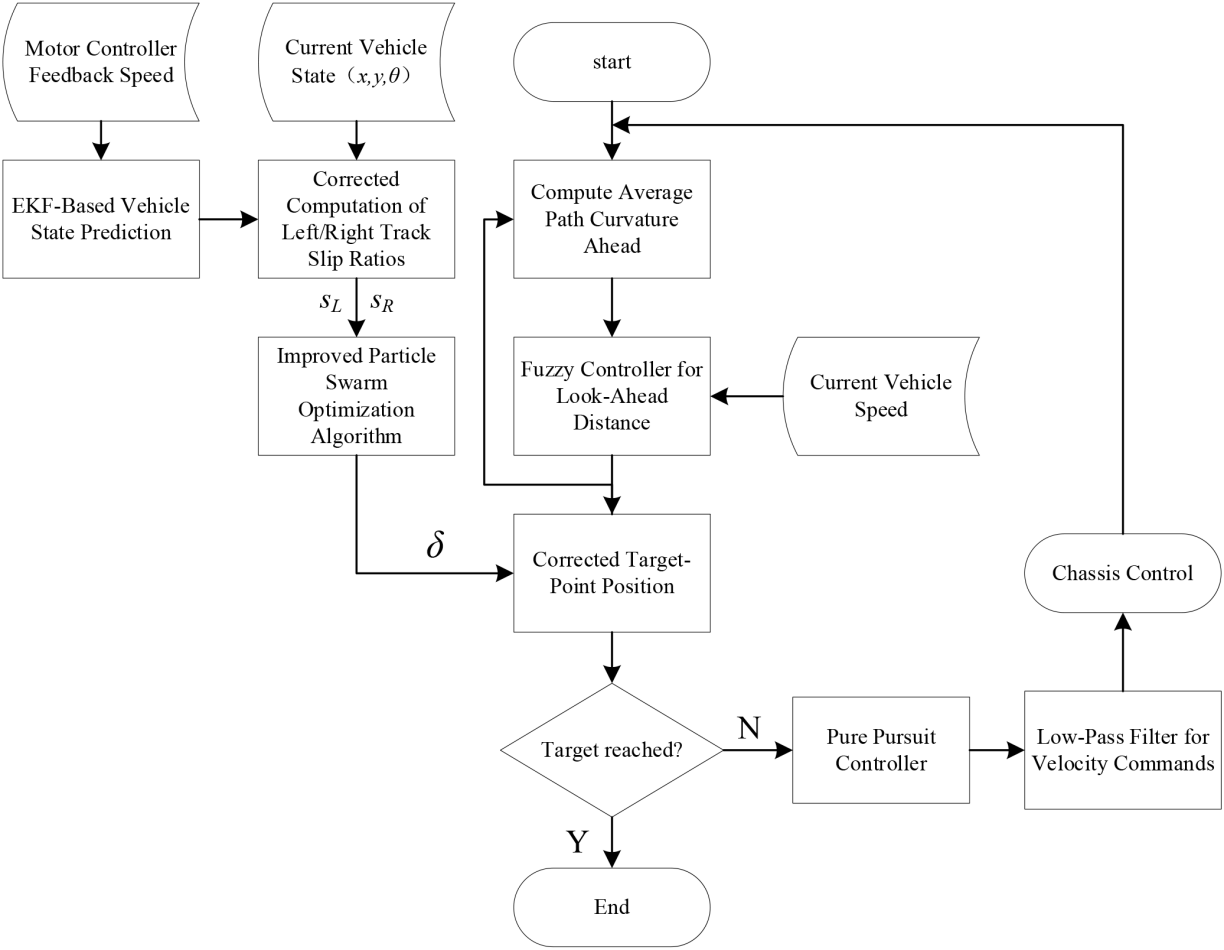
Overall flowchart of the control system.

The proposed system integrates multiple modules, including an extended Kalman filter, a fuzzy controller, an improved particle swarm optimization algorithm, and a Pure Pursuit controller. The overall control flow is shown in **Figure 3**. First, the fuzzy controller takes the curvature of the reference path ahead and the vehicle speed as inputs to determine an appropriate look-ahead distance. Then, the extended Kalman filter estimates the slip ratios of the left and right tracks, *s_L_*"and *s_R_*, based on the vehicle states and the motor speed feedback from the motor controller. Next, the improved particle swarm optimization algorithm uses this information to compute the optimal offset angle *δ* for the current look-ahead point. The Pure Pursuit algorithm then calculates the velocities of the left and right tracks according to the corrected look-ahead point, and a low-pass filter is applied to suppress unnecessary oscillations. Finally, the commanded velocities of the left and right tracks are sent to the chassis actuators, forming a closed-loop perceive–optimize–control process that continues until the look-ahead point reaches the end of the reference path, at which point the procedure terminates.

**Figure 4:**
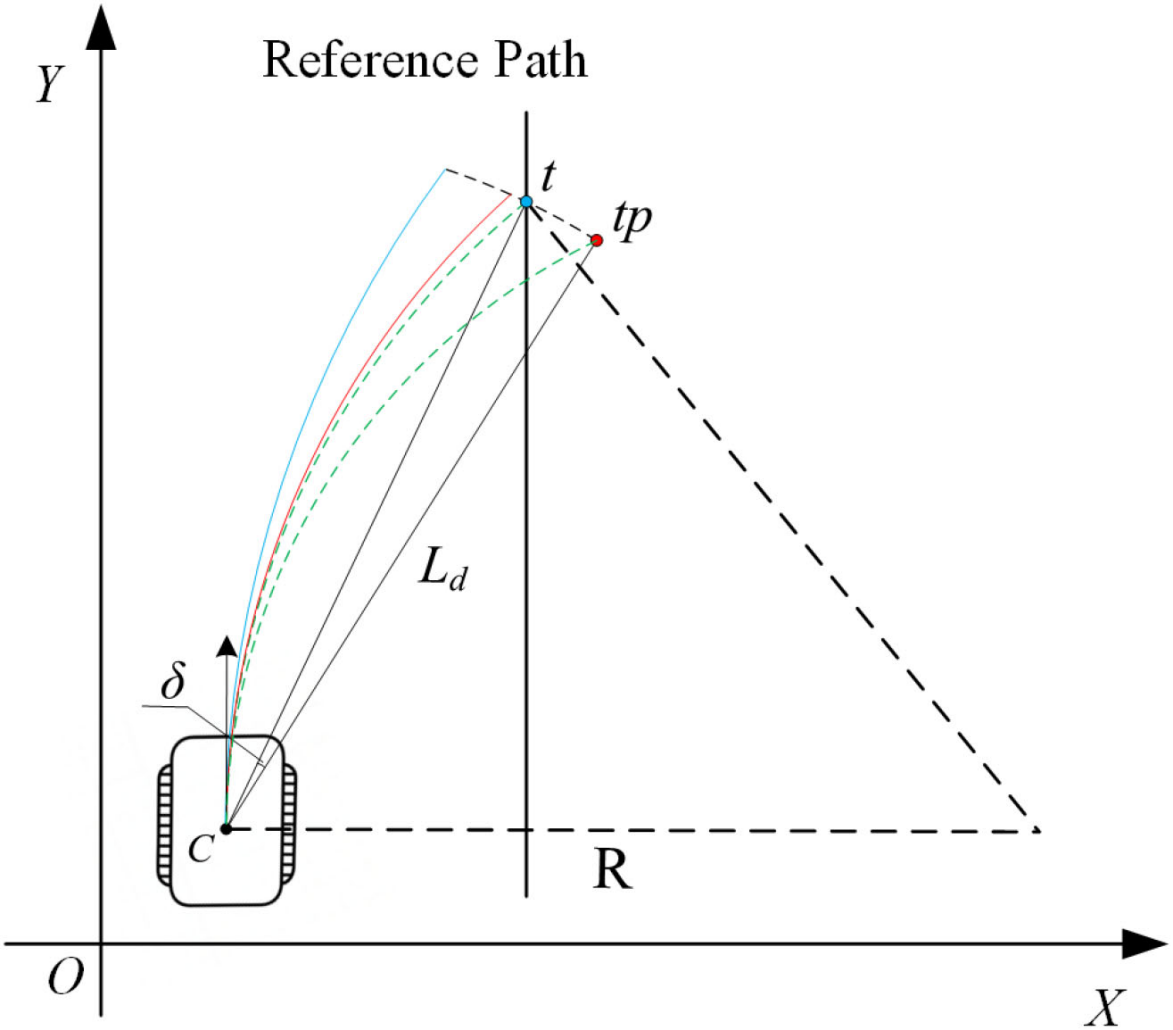
Schematic of target point offset and trajectory response under slip compensation.

To further illustrate the role of the look-ahead target point offset compensation mechanism under track slip conditions, **Figure 4** presents the trajectory variations under different control strategies. In the figure, the green line represents the ideal vehicle trajectory, the blue line indicates the trajectory with slip-induced deviation, and the red line shows the actual trajectory obtained after applying the target-point offset *δ*. It can be clearly observed that by adjusting the target point, the controller is indirectly guided to generate velocity commands that better conform to the theoretical trajectory, thereby improving the vehicle’s adaptability to track slip.

## 3 Design of key algorithms

### 3.1 Adaptive look-ahead distance strategy based on fuzzy control

In Pure Pursuit–based path tracking control, the selection of the look-ahead distance *L_d_* plays a crucial role in determining control accuracy and system stability (Chen et al., 2024). To meet the dynamic control requirements under varying speeds and path geometric complexities, an adaptive look-ahead distance adjustment mechanism based on fuzzy control is designed. This mechanism enables automatic tuning of *L_d_*​ during vehicle operation, thereby enhancing the system’s environmental adaptability and control robustness.

**Figure 5:**
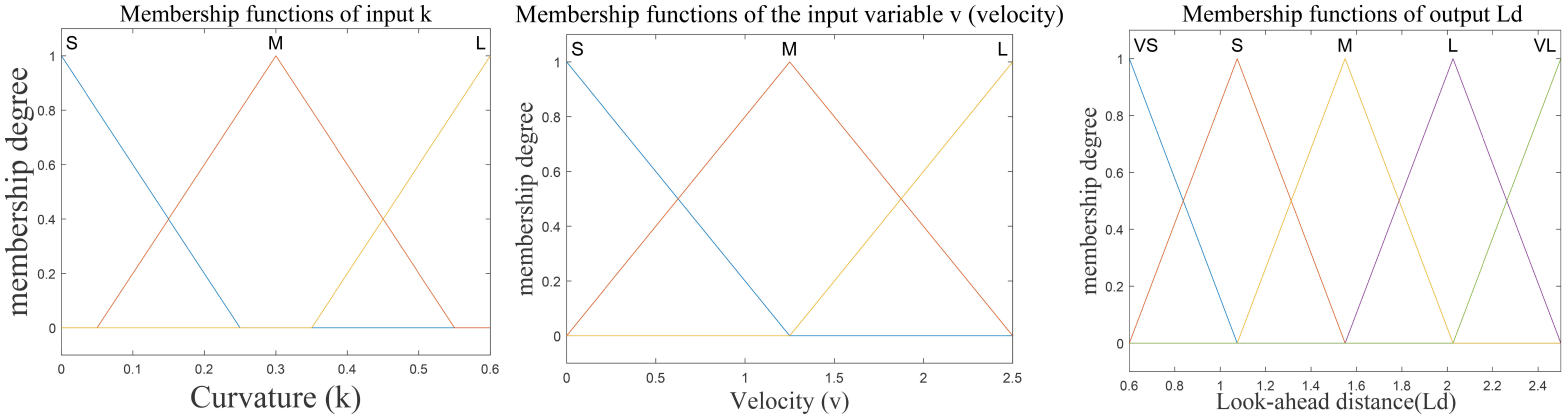
Distribution diagram of membership functions.

The designed fuzzy controller adopts a two-input, single-output structure. The input variables include the current linear velocity *v* of the vehicle, defined within the range [0, 2.5] and divided into three levels, and the average curvature *k* of the path ahead, defined within the range [0, 0.6] and also divided into three levels. The output variable is the adaptive look-ahead distance L_d_, with an output range of [0.6, 2.5] and divided into five levels. All variables are represented using triangular membership functions, as shown in **Figure 5**.

The velocity range [0, 2.5] was selected according to the maximum operating speed of the test platform in both simulation and field experiments. The curvature range [0, 0.6] was determined based on the curvature statistics of the reference path, which covers the typical turning radii observed in agricultural trajectories. These ranges ensure that the fuzzy controller operates within the actual dynamic limits of the vehicle.

**Figure 6:**
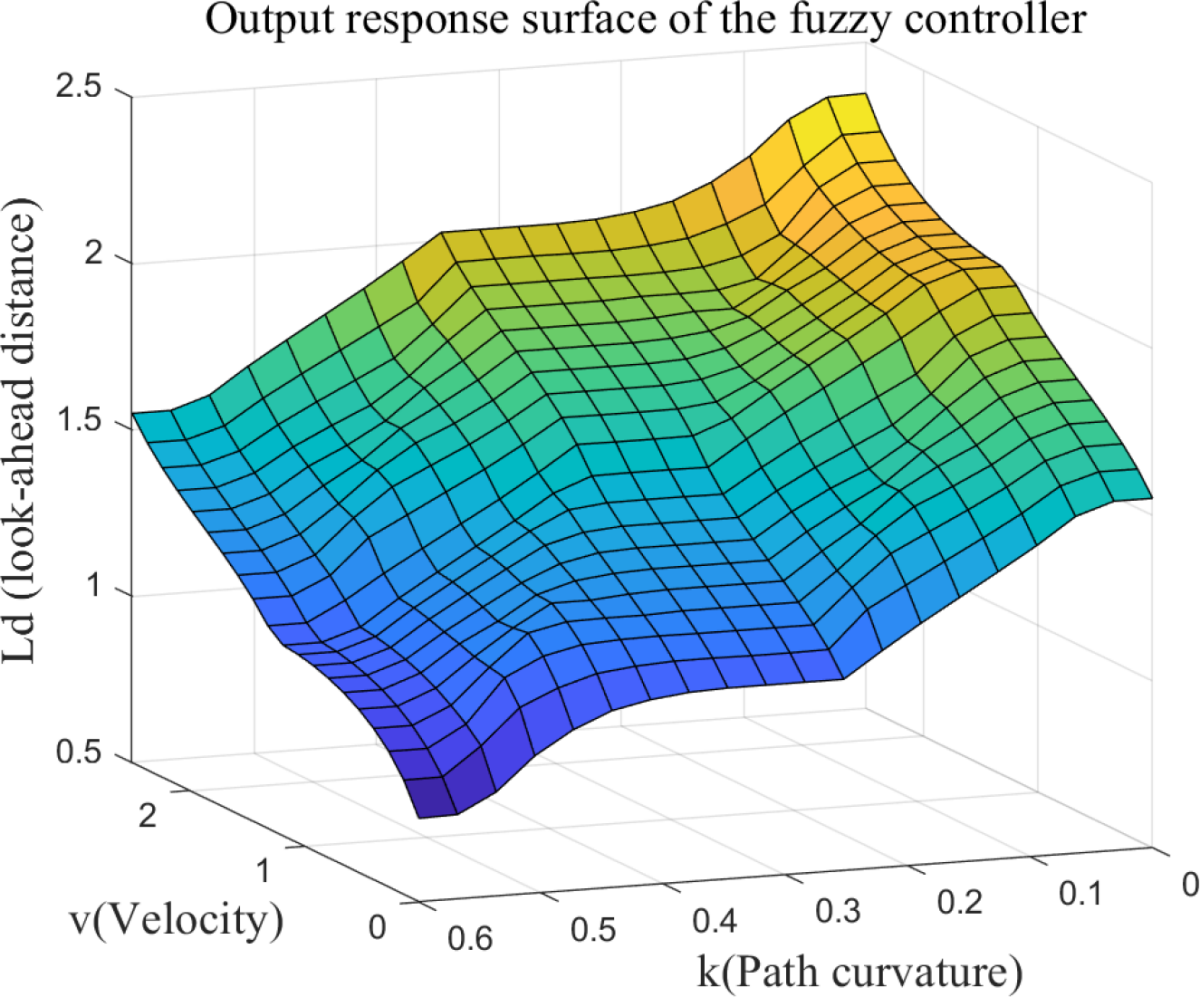
Surface of fuzzy control rules.

Based on the response characteristics of path tracking, nine fuzzy control rules are designed. The fuzzy rule table is shown in **Table 1**, and the corresponding rule surfaces are illustrated in **Figure 6**.

**Table 1:** Fuzzy control rules.

V	K		
	S	M	L
S	M	S	VS
M	L	M	S
L	VL	L	M

### 3.2 Design of the slip-aware target-point offset mechanism

To realize the slip-aware target-point offset mechanism, the key lies in accurately acquiring slip information during vehicle operation and adjusting the control strategy accordingly. The mechanism consists of two main components. First, an extended Kalman filter (EKF) is employed to estimate the slip ratios of the left and right tracks in real time, which serve as critical indicators of ground adhesion conditions. Second, guided by the difference in slip ratios, a target-point offset angle *δ* is introduced. An objective function is constructed based on this slip information, and an improved particle swarm optimization (PSO) algorithm—enhanced with a Chebyshev-window-based weight attenuation strategy—is utilized to iteratively obtain the optimal offset angle *δ* for the current operating condition.

#### 3.2.1 Estimation of left and right track slip ratios based on the extended Kalman filter

To achieve online estimation of the slip ratios of the left and right tracks, a state estimation algorithm based on the extended Kalman filter (EKF) is designed. This method considers the slip characteristics of tracked agricultural machinery by fusing pose measurements from RTK-GNSS and the IMU with the commanded track velocities. A nonlinear state transition model is constructed, and the slip ratios are inferred through the observation residuals, thereby enabling slip-state perception without requiring additional sensors.

The system state vector is defined as follows, as given in Equation 11:

(11) $$\chi ={\left[x,y,\theta ,s_{L},s_{R}\right]}^{T}$$

where *x* and *y* represent the position of the vehicle’s center of mass in the global coordinate frame, *θ* denotes the heading angle, and *s_L_* and *s_R_* are the slip ratios of the left and right tracks, respectively.

The control inputs are obtained from the motor controller in the form of the rotational speeds of the left and right drive motors, denoted as *n_L_*"and *n_R_* (unit: r/min). The motor speeds are transmitted to the tracks through the vehicle’s reduction mechanism and the drive sprockets, generating the corresponding track linear velocities. According to the relationship between the wheel rotational speed and the track linear velocity, the ideal linear velocities of the left and right tracks are given by Equation 12:

(12) $$\left\{ \begin{array}{l} v_{L}^{ideal}=\frac{2\pi r}{60i}\cdot n_{L}\\ v_{R}^{ideal}=\frac{2\pi r}{60i}\cdot n_{R} \end{array}\right.$$

where *r* is the radius of the drive sprocket, and *i* is the transmission ratio between the motor shaft and the drive sprocket.

Therefore, the control input vector of the extended Kalman filter is given by Equation 13:

(13) $$u={\left[v_{L}^{ideal},v_{R}^{ideal}\right]}^{T}$$

The filter predicts the vehicle’s current position and orientation by combining the previous state estimate with the wheel-speed information through the nonlinear kinematic model, thereby providing a reasonable prior state estimate for the update step.

Considering the influence of slip on the actual track velocities, the actual velocities of the left and right tracks can be expressed as Equation 14:

(14) $$\left\{ \begin{array}{l} v_{L}^{real}=(1-s_{L,k-1})\cdot v_{L,k-1}^{ideal}\\ v_{R}^{real}=(1-s_{R,k-1})\cdot v_{R,k-1}^{ideal} \end{array}\right.$$

Accordingly, the actual linear velocity of the vehicle’s center of mass and its angular velocity can be expressed as shown in Equation 15:

(15) $$\left\{ \begin{array}{l} v_{k-1}^{real}=\frac{v_{L}^{real}+v_{R}^{real}}{2}\\ \omega_{k-1}^{real}=\frac{v_{R}^{real}-v_{L}^{real}}{b} \end{array}\right.$$

Substituting these expressions into the prediction model yields the state prediction at time step k as given in Equation 16:

(16) $$\{\begin{array}{c} {\hat{x}}_{k|k-1}={\hat{x}}_{k-1}+v_{k-1}^{real}\cdot \cos({\hat{\theta }}_{k-1})\cdot dt\\ {\hat{y}}_{k|k-1}={\hat{y}}_{k-1}+v_{k-1}^{real}\cdot \sin({\hat{\theta }}_{k-1})\cdot dt\\ {\hat{\theta }}_{k|k-1}={\hat{\theta }}_{k-1}+\omega_{k-1}^{real}\cdot dt\\ {\hat{S}}_{L,k|k-1}={\hat{S}}_{L,k-1}\\ {\hat{S}}_{R,k|k-1}={\hat{S}}_{R,k-1} \end{array}$$

In this formulation, the slip ratios are treated as slow-varying variables and are therefore not updated during the prediction step; instead, they are corrected only during the measurement update phase.

By linearizing the state transition function with respect to the state variables, the Jacobian matrix F is obtained. With the process noise covariance denoted as Q, the predicted state covariance can be expressed as Equation 17:

(17) $$P_{k|k-1}=F_{k}P_{k-1}F_{k}^{T}+Q$$

After completing the state prediction, the extended Kalman filter updates the predicted state using the sensor measurements at the current time step. By comparing the predicted values with the actual observations (i.e., the measurement residual) and incorporating the uncertainty information represented by the covariance matrices, the filter computes the optimal state estimate. This process enables dynamic tracking of the vehicle’s actual state.

The observation vector of the extended Kalman filter is provided by the fusion of RTK-GNSS and IMU measurements, and includes the vehicle’s two-dimensional position and heading angle in the global coordinate frame, as shown in Equation 18:

(18) $$z_{k}={\left[x_{k}^{abs},y_{k}^{abs},\theta_{k}^{abs}\right]}^{T}$$

The observation model can be expressed as Equation 19:

(19) $$z_{k}=H\cdot \chi_{k}+v_{k}$$

where *v_k_*"is the measurement noise, assumed to follow a zero-mean Gaussian distribution with covariance *R*. The observation matrix H is defined as shown in Equation 20:

(20) $$H=[\begin{array}{ccccc} 1 & 0 & 0 & 0 & 0\\ 0 & 1 & 0 & 0 & 0\\ 0 & 0 & 1 & 0 & 0 \end{array}]$$

The measurement residual is constructed from the difference between the current predicted observation and the actual measurement at time step, as given in Equation 21:

(21) $${\tilde{z}}_{k}=z_{k}-H\cdot {\hat{\chi }}_{k|k-1}$$

where the residual of the heading angle requires angle normalization, as shown in Equation 22:

(22) $${\tilde{z}}_{k}^{\left(\theta \right)}=wrapToPi(\theta_{k}^{obs}-{\hat{\theta }}_{k|k-1})$$

The Kalman gain is computed as given in Equation 23:

(23) $$K_{k}\;=P_{k|k-1}H^{T}\;{\left({\text{HP}}_{k|k-1}H^{T}\;+\;R\right)}^{-1}$$

The state estimate is updated as shown in Equation 24:

(24) $${\hat{\chi }}_{k}\;={\hat{\chi }}_{k|k-1}+K_{z}\cdot {\tilde{z}}_{z}$$

The covariance matrix is updated as shown in Equation 25:

(25) $$P_{k}=(I-K_{k}H)P_{k|k-1}$$

The process noise matrix Q and measurement noise matrix R were determined according to the nominal specifications of the RTK–IMU sensor and refined through empirical tuning. R was initialized based on the RTK–IMU accuracy (2–3 *cm* in position and about 0.2*°* in heading), while Q was selected to represent slip variation and model uncertainty, and was adjusted through repeated simulations and field tests to ensure stable EKF convergence.

#### 3.2.2 Improved particle swarm optimization algorithm based on the chebyshev window

To enhance the adaptive adjustment performance of the slip compensation angle *δ* in Pure Pursuit control, this study employs a particle swarm optimization (PSO) algorithm to rapidly compute the target offset angle *δ* within each control cycle. The PSO method offers advantages such as simple structure, strong global search capability, and fast convergence, making it well suited for single-variable real-time optimization problems in trajectory tracking tasks (Sun et al., 2022).

It is also worth noting that PSO-based parameter optimization has been widely adopted in mobile and tracked robot control using simplified kinematic models. Yang et al. applied an improved PSO algorithm to optimize the controller of a crawler robot while relying on a non-dynamic model, and achieved high tracking accuracy in unstructured environments (Yang et al., 2025). This further supports the adequacy of using a kinematic model for optimization in low-speed tracked platforms.

PSO simulates the cooperative search behavior of a particle swarm (such as a flock of birds or a school of fish) in the solution space. Suppose the swarm contains N particles, and the position and velocity of each particle are represented as shown in Equation 26:

(26) $$\left\{ \begin{array}{c} X_{i}={\left[x_{i1},x_{i2},\ldots ,x_{id}\right]}^{T}\\ V_{i}={\left[v_{i1},v_{i2},\ldots ,v_{id}\right]}^{T} \end{array}\right.$$

The update equations for the i-th particle at iteration t are given by Equation 27:

(27) $$\left\{ \begin{array}{l} v_{i}^{\left(t+1\right)}=\omega \cdot v_{i}^{\left(t\right)}+c_{1}r_{1}(p_{i}-x_{i}^{\left(t\right)})+c_{2}r_{2}(p_{i}-x_{i}^{\left(t\right)})\\ x_{i}^{\left(t+1\right)}=x_{i}^{\left(t\right)}+v_{i}^{\left(t+1\right)} \end{array}\right.$$

where *p_i_*"is the personal best position of the *i*-th particle, *g* is the global best position of the swarm, *c_1_*"and *c_2_*"are the cognitive and social learning factors, respectively, *r_1_,r_2_~U(0,1)* are uniformly distributed random numbers, and *ω* is the inertia weight used to balance global exploration and local convergence.

To compensate for the heading deviation caused by asymmetric slip between the left and right tracks, a slip compensation angle *δ* is introduced to adjust the direction of the target point. The controller adopts a “slip-aware look-ahead point offset” strategy, meaning that an optimal offset angle is selected in each control cycle to minimize the deviation between the actual vehicle trajectory and the ideal trajectory over a future prediction horizon.

Let the current state be (x, y, θ), the slip ratios estimated by the EKF be *s_L_*"and *s_R_*, and the reference path be denoted as *Path*. The multi-step prediction cost function is constructed as shown in Equation 28:

(28) $$J(\delta )=\sum_{k=1}^{N_{step}}\left[\omega_{pos}\cdot ∥X_{k}^{real}(\delta )-X_{k}^{\text{ideal}}{∥}^{2}+\omega_{yaw}\cdot {\left(\theta_{k}^{real}(\delta )-\theta_{k}^{\text{ideal}}\right)}^{2}\right]$$

where $X_{k}^{real}(\delta )$ represents the actual vehicle position at step k considering slip and the offset angle *δ*, and $X_{k}^{ideal}$ denotes the ideal position under the no-slip and no-offset condition. N_step_ is the prediction horizon, and *ω_pos_* and *ω_yaw_*"are the weights for the position and heading errors, respectively. In this study, *ω_pos_* = 1.0 and *ω_yaw_* = 0.2.

The weights *ω_pos_* = 1.0 and *ω_yaw_* = 0.2 were chosen according to the relative impact of lateral and heading errors on the offset angle *δ*. A higher *ω_pos_* ensures effective correction of lateral deviation, while a smaller *ω_yaw_* avoids excessive steering adjustments. Preliminary tuning showed that this ratio achieves stable and smooth compensation, and δ remains insensitive within a reasonable range of weight variations.

The optimization objective is to minimize this cost function using the particle swarm optimization algorithm, as expressed in Equation 29:

(29) $$\delta^{*}=\arg\underset{\delta \in [-\delta_{\max},\delta_{\max}]}{\min}J(\delta )$$

The optimization process of the particle swarm algorithm is as follows:

1. Population initialization: Randomly initialize particle positions and velocities within the interval δ∈[−3π​,3π​].
2. Fitness evaluation: Each particle corresponds to a candidate offset angleδ; the multi-step prediction function is executed to compute the objective function *J(δ)*.
3. Position update: Particle positions are iteratively updated according to the update equations.
4. Termination: The algorithm terminates when the maximum number of iterations is reached or when the cost function converges, and the optimal δ^*^ is output.

The inertia weight *ω* controls the degree to which the current velocity of a particle is preserved and is a key factor influencing PSO convergence performance. A larger *ω* favors global exploration, whereas a smaller*ω*strengthens local search capability. Traditional approaches commonly adopt a linear decreasing inertia weight strategy, as given in Equation 30:

(30) $$\omega^{\left(t\right)}=\omega_{\max}-\frac{\omega_{\max}-\omega_{\min}}{T}\cdot t$$

However, this setting provides limited exploration capability in the early iterations of the particle swarm, making it prone to local optima. Moreover, the linear decay of the inertia weight causes a slow reduction in the later stages, which may result in excessively large particle velocities near convergence, leading to oscillation and unstable convergence behavior (/Arasomwan et al., 2013). To enhance early-stage exploration and late-stage convergence, this study introduces a nonlinear decay strategy with a Chebyshev-like window shape (Guo and Wu, 2018), as expressed in Equation 31:

(31) $$\omega^{\left(t\right)}=\omega_{\min}+(\omega_{\max}-\omega_{\min})\cdot \cos^{K}(\frac{\pi t}{2T})$$

where t denotes the current iteration number and T is the maximum number of iterations. *ω_max_*"and *ω_min_* represent the initial and final inertia weights, respectively. The parameter *K>0* controls the nonlinearity of the decay curve; *K=1* corresponds to the standard cosine attenuation, while *K<1* produces a smoother curve that emphasizes exploration. Based on multiple trials in this study, setting *K=0.8* allows the algorithm to maintain strong global search capability in the early stage while ensuring rapid convergence in the later stage, thereby improving optimization accuracy.

**Figure 7:**
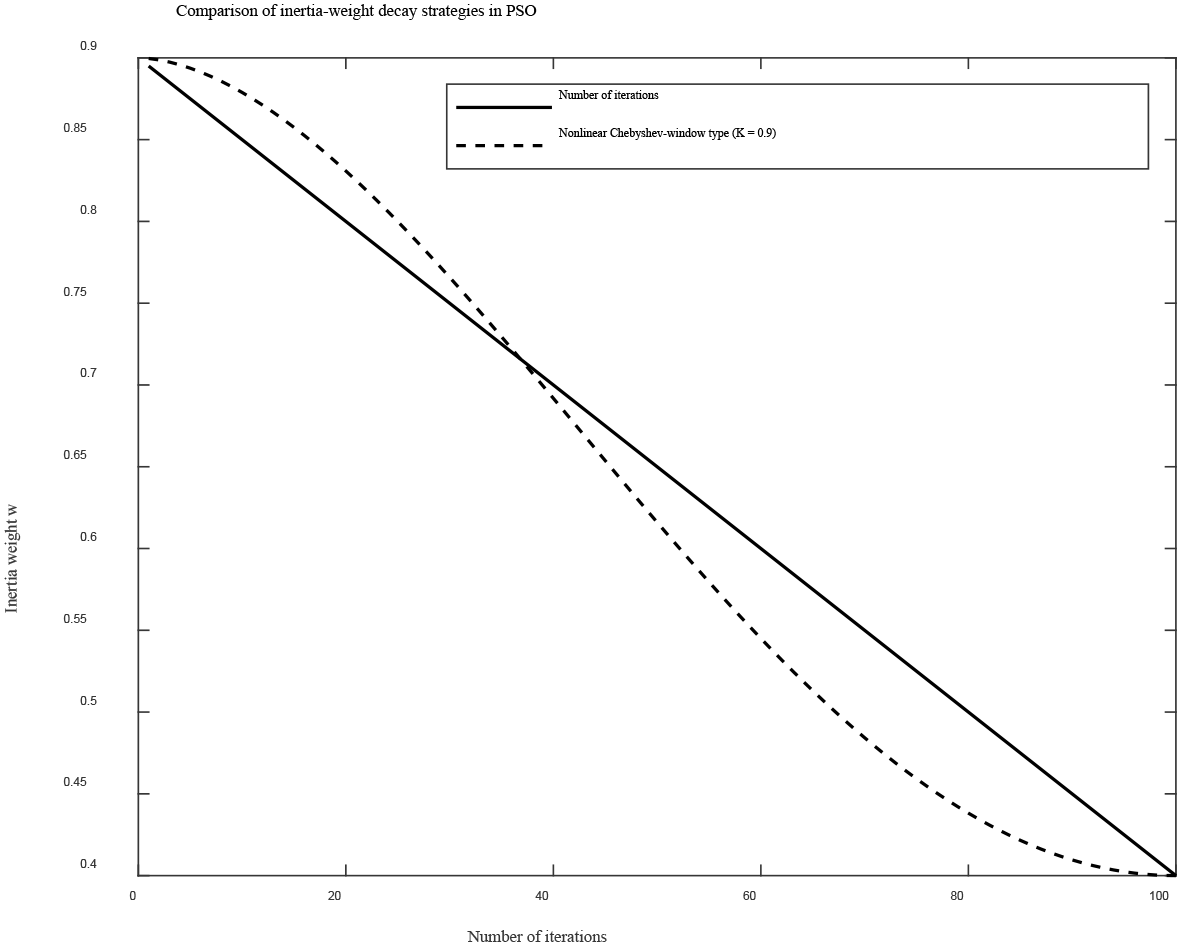
Comparison of variation trends under different inertia weight decay strategies.

As shown in **Figure 7**, the curves of the inertia weight under linear decay and the proposed Chebyshev-like nonlinear decay are compared. It can be observed that the improved inertia-weight curve preserves the “memory” of the particle swarm during the early iterations, enabling broader exploration of the global solution space, while the rapid decay in the later stage enhances local search capability and solution refinement.

Multiple comparative simulation experiments were conducted in the MATLAB R2022b environment using the two inertia-weight strategies. The swarm size was set to 30, the maximum number of iterations to 100, and the control-cycle compensation interval to 0.1 s, with the optimization of *δ* performed once per cycle.

The results are summarized in **Table 2**. The experimental results show that the nonlinear decay strategy with a Chebyshev-like window significantly reduces the average computation time compared with the linear decay strategy, while also providing slightly improved solution accuracy. The average lateral error is reduced by 5.8%.

**Table 2:** Performance comparison under different inertia weight decay strategies.

Inertia strategy	Initial value *ω_max_*	Final value *ω_min_*	Control factor K	Average computation time(ms)	Average lateral error(cm)
Average lateral error	0.9	0.4	/	49.81	6.18
Chebyshev-like	0.9	0.4	0.8	26.74	5.82

### 3.3 Filtering of velocity command signals

To suppress sensor noise and high-frequency oscillations generated within the controller, a first-order low-pass filter is introduced to smooth the commanded velocities of the left and right tracks (Pagkalos et al., 2023). Its discrete-time formulation is expressed as shown in Equation 32:

(32) $$\left\{ \begin{array}{l} v_{L}^{\left(k\right)}=\beta \cdot v_{L}^{\left(k-1\right)}+(1-\beta )\cdot {\hat{v}}_{L}^{\left(k-1\right)}\\ v_{R}^{\left(k\right)}=\beta \cdot v_{R}^{\left(k-1\right)}+(1-\beta )\cdot {\hat{v}}_{R}^{\left(k-1\right)} \end{array}\right.$$

where ${\hat{v}}_{L}^{\left(k-1\right)}$ and ${\hat{v}}_{R}^{\left(k-1\right)}$ are the calculated left and right track velocities at time step k; $v_{L}^{\left(k\right)}$ and $v_{R}^{\left(k\right)}$ are the filtered velocities used as the actual motor command inputs; and β∈[0,1] is the filtering coefficient, where a larger value results in smoother filtering with slower response, while a smaller value yields a more responsive output.

In this study, *β* = 0.6 is empirically selected, which effectively suppresses fluctuations in the commanded velocities without causing significant response delay, thereby improving the stability of track actuation and the accuracy of path tracking.

## 4 Simulation and experimental results analysis

### 4.1 Simulation validation

**Figure 8:**
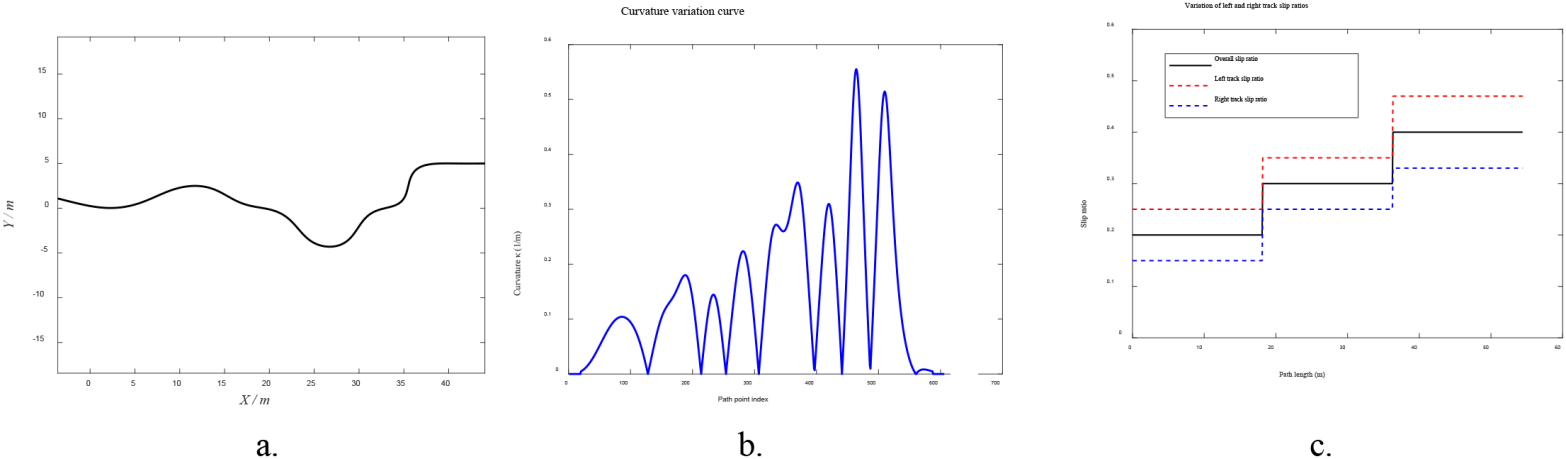
Schematic of path and slip condition settings. **(a)** Reference path diagram, **(b)** Path curvature variation curve, **(c)** Left and right track slip ratio variation diagram.

To verify the effectiveness of the proposed slip-aware look-ahead point offset path tracking method, simulation experiments were first conducted using MATLAB R2022b. The simulation model was constructed based on the differential-drive kinematic equations of the tracked agricultural vehicle, with slip disturbances introduced to emulate non-ideal operating conditions in complex farmland terrain. The main simulation parameters are set as follows: track width of 0.5 m, operating speed of 0.75 m/s, and sampling period of 0.1 s. The reference path, as shown in **Figure 8a**, has a total length of approximately 40 m and exhibits an “S-shaped” profile. The corresponding curvature distribution in **Figure 8b** demonstrates its ability to represent complex terrain trajectories encountered in farmland operations. To simulate the challenging conditions of soft farmland soil, segmentally varying slip ratios were introduced, as illustrated in **Figure 8c**. The slip ratios of both tracks exhibit a step-wise increasing pattern, with the left track exhibiting a generally higher slip level than the right track. The combined slip ratio eventually reaches approximately 0.4, providing the necessary basis for validating the slip compensation capability of the proposed control strategy.

Two control methods were compared in the simulation experiments:(1) the traditional fuzzy Pure Pursuit control; and (2) the proposed improved method integrating EKF-based slip estimation and Chebyshev-window-enhanced PSO compensation.

It should be emphasized that the objective of this work is not to redesign a completely new dynamic controller, but to enhance the slip robustness of geometric path-tracking schemes that are widely deployed on low-speed tracked agricultural platforms. Therefore, a fuzzy Pure Pursuit controller is selected as a representative baseline method due to its simple structure, low implementation cost, and strong real-time performance. The proposed slip-aware offset compensation is integrated on top of this baseline to improve tracking accuracy under slip disturbances.

**Figure 9:**
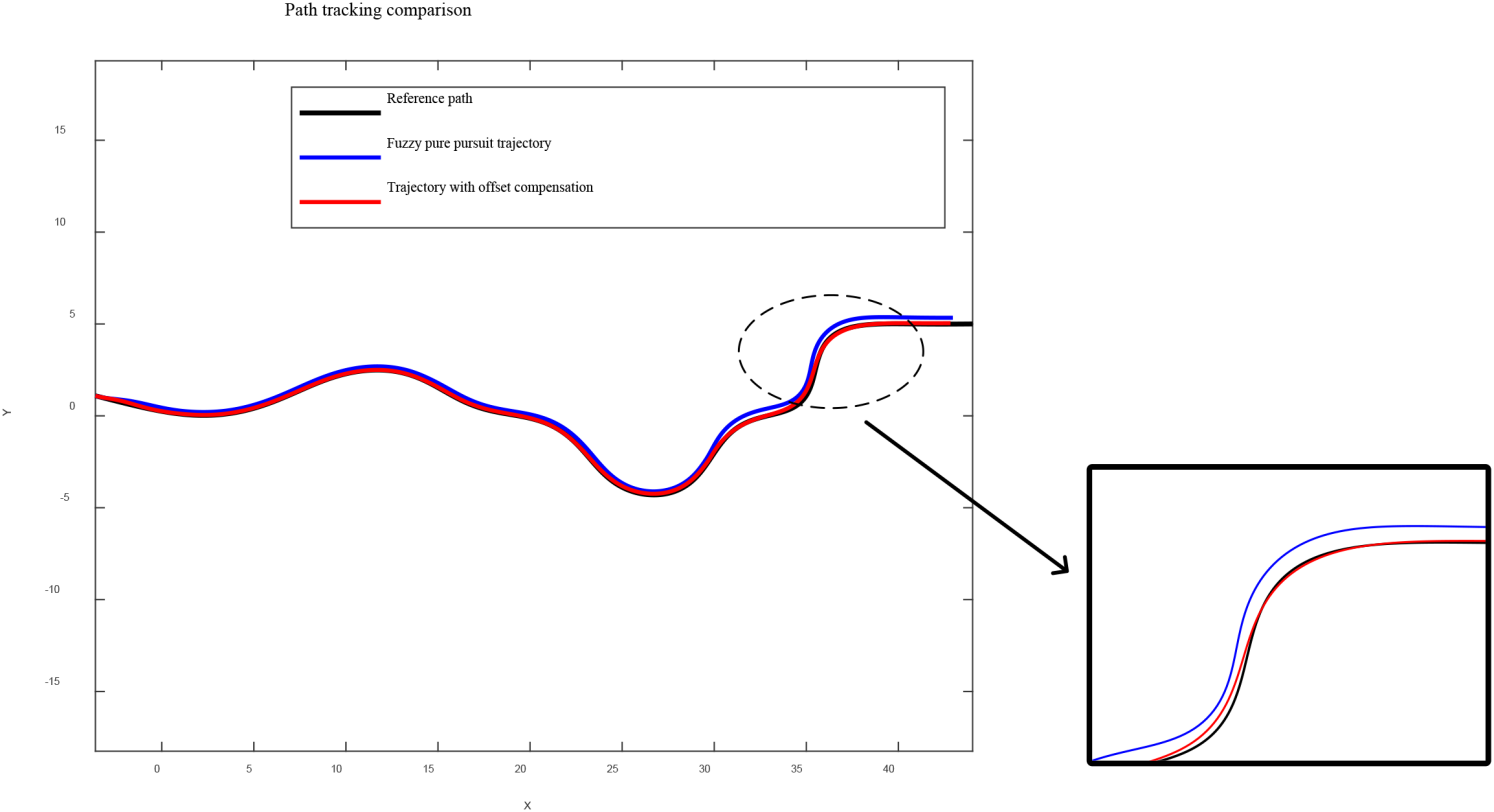
Path-tracking comparison results (including locally enlarged view).

**Figure 10:**
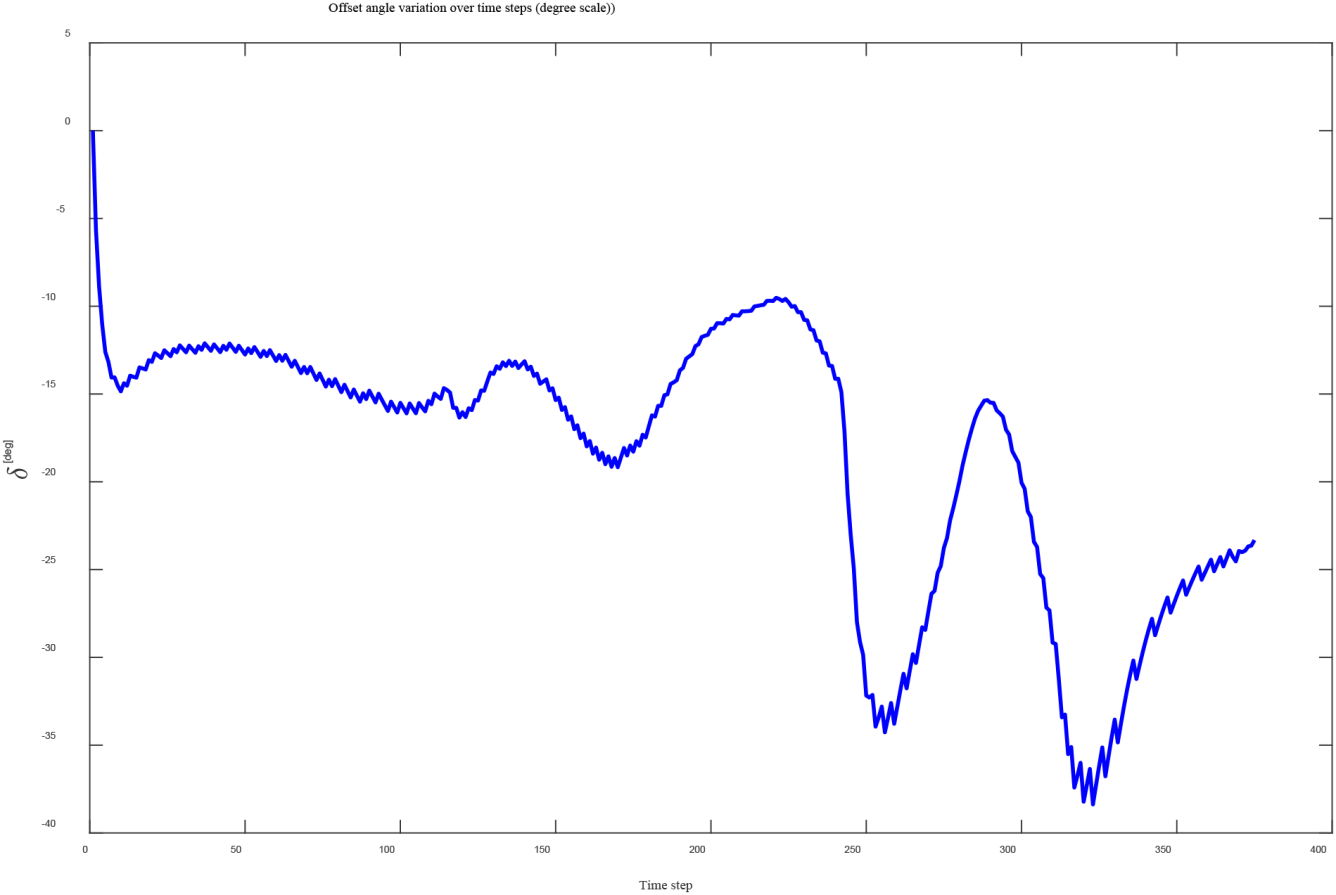
Variation curve of offset compensation angle with time steps.

The trajectory comparison results for the “S-shaped” path are shown in **Figure 9**. Under the traditional fuzzy Pure Pursuit control, significant trajectory deviation occurs when slip is present, with an average lateral error of 0.26 m. The lateral deviation further increases in regions with large curvature variations, reaching a maximum deviation of 0.41 m.In contrast, the proposed method compensates for the slip-induced heading deviation by adjusting the target-point offset angle in real time according to the estimated slip ratios. The real-time evolution of the offset angle is shown in **Figure 10**. After applying the slip-aware offset compensation, the actual trajectory closely follows the ideal path, with an average lateral error of 0.05 m and a maximum lateral error of 0.12 m.

The simulation results demonstrate that the proposed path tracking algorithm maintains high tracking accuracy even under significant slip conditions.

### 4.2 Field experiment

**Figure 11:**
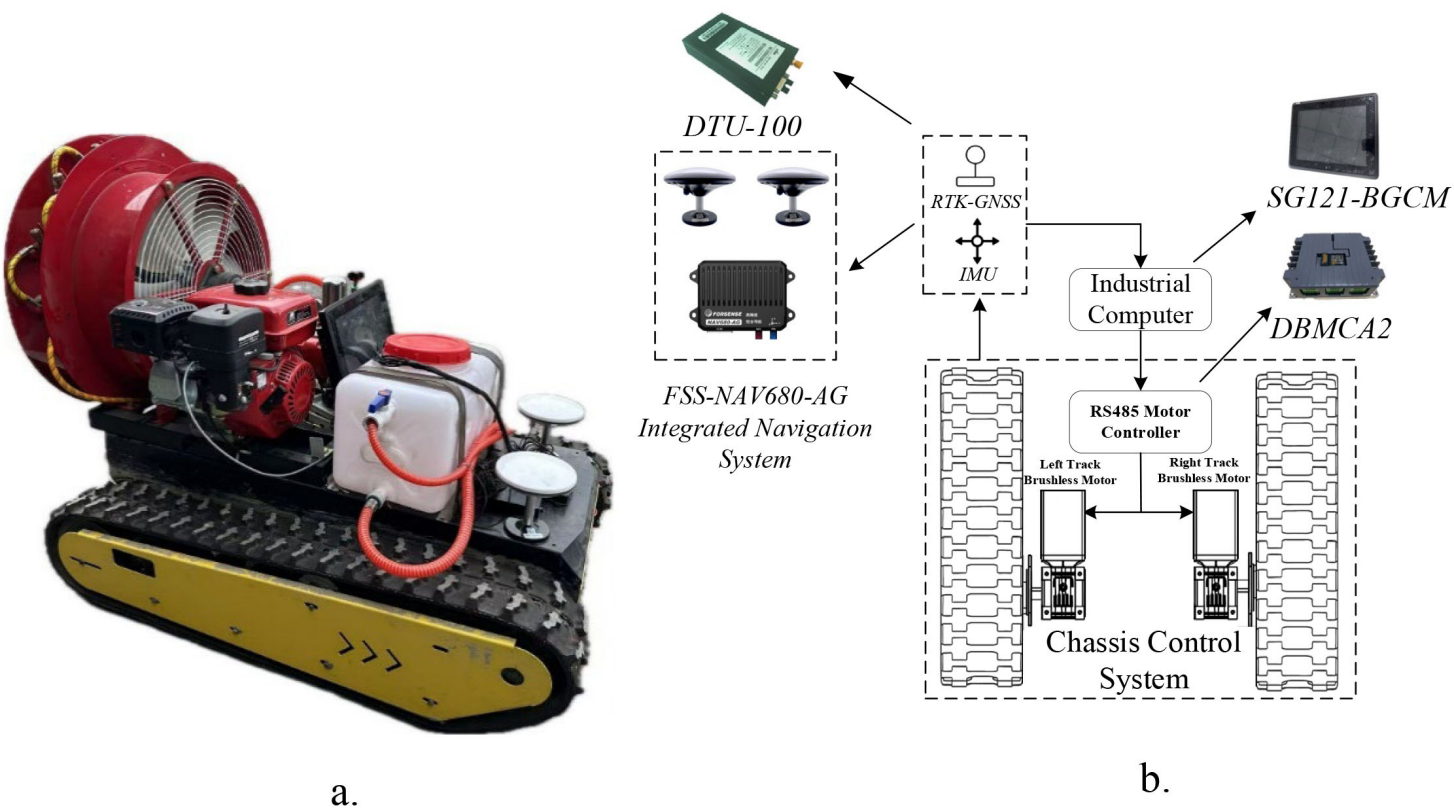
Path-tracking experimental platform of tracked agricultural vehicle and composition of the control system. **(a)** Tracked agricultural vehicle (real machine), **(b)** Control system composition diagram.

To further validate the effectiveness of the proposed path tracking algorithm, a field experiment platform based on a tracked chassis was constructed. The physical structure and system architecture of the platform are shown in **Figure 11**. The experimental platform mainly consists of a main control system, a drive system, a positioning module, and an operation module.

The test vehicle adopts a tracked differential-drive configuration. A chemical tank is mounted at the front of the chassis, while the rear is equipped with a diesel-powered air-assisted sprayer, enabling pesticide spraying functions required for plant protection operations. The left and right tracks are independently driven by brushless DC motors coupled with reduction gearboxes, enabling differential steering and straight-line motion. Before each experiment, the RTK–IMU module was initialized using the default zero-bias settings, followed by a short static alignment procedure to ensure coordinate consistency between the RTK and IMU outputs.

The main parameters of the vehicle are listed in **Table 3**.

**Table 3:** Main parameters of the tracked agricultural vehicle experimental platform.

Item	Value
Vehicle type	Tracked differential-drive chassis
Vehicle dimensions	1.30m×0.80m×0.40m(L × W × H)
Track width	0.15m
Track center distance	0.65m
Reduction ratio	40
Maximum motor speed	4000 RPM
Operation device	Diesel-powered sprayer

**Figure 12:**
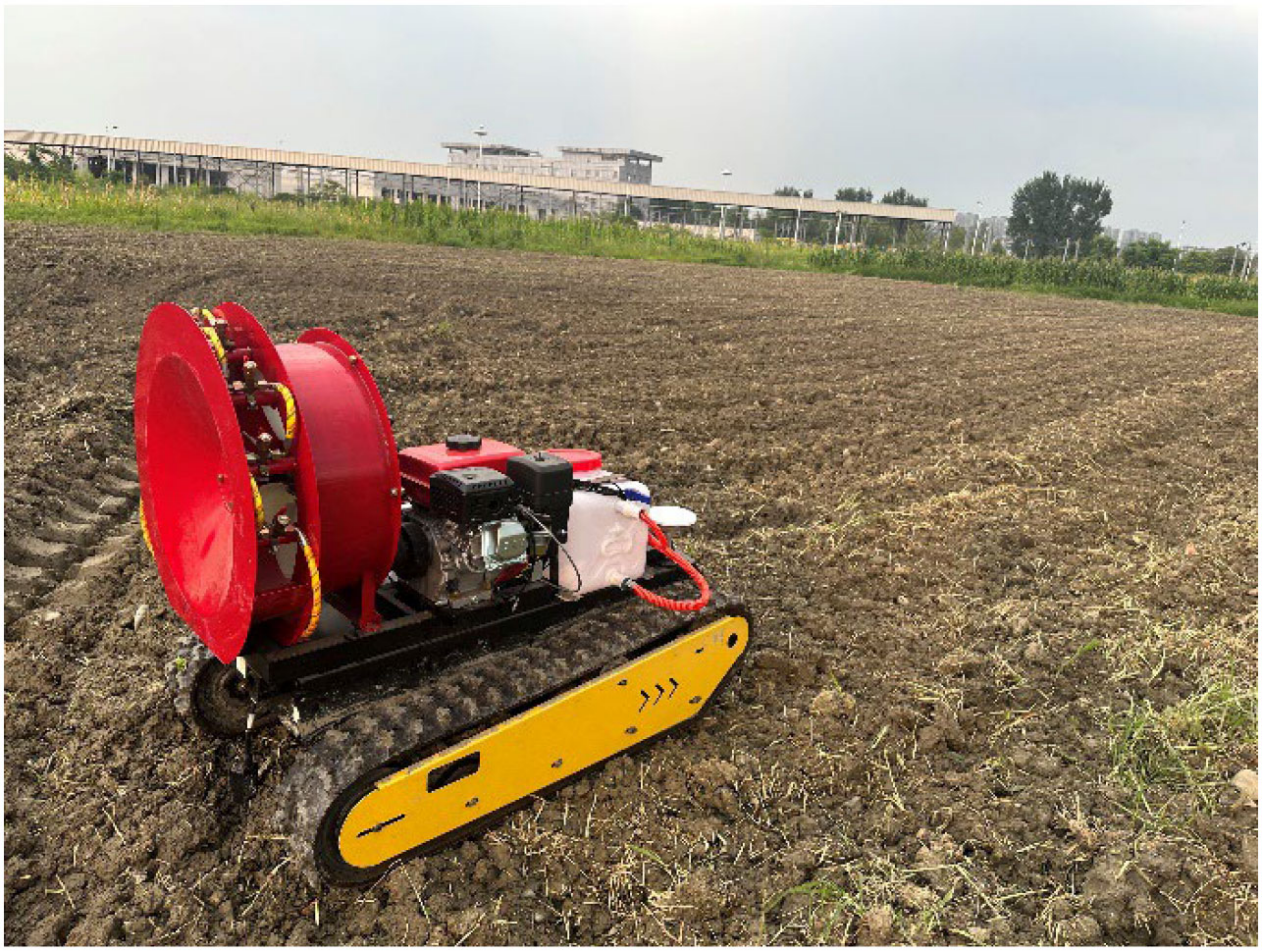
Real-vehicle experiment of navigation tracking.

The field experiments were conducted in the test farmland at the Pidu Campus of Xihua University, as shown in **Figure 12**. The ground surface was in a cultivated state with soft soil conditions, representing a typical high-slip terrain. The experimental trajectory was designed as a loop-shaped path consisting of straight segments and turning segments, with turning radii ranging from 2.5 m to 5 m. This layout effectively evaluates the adaptability, robustness, and slip-awareness capability of the proposed path tracking algorithm under complex terrain conditions.

**Figure 13:**
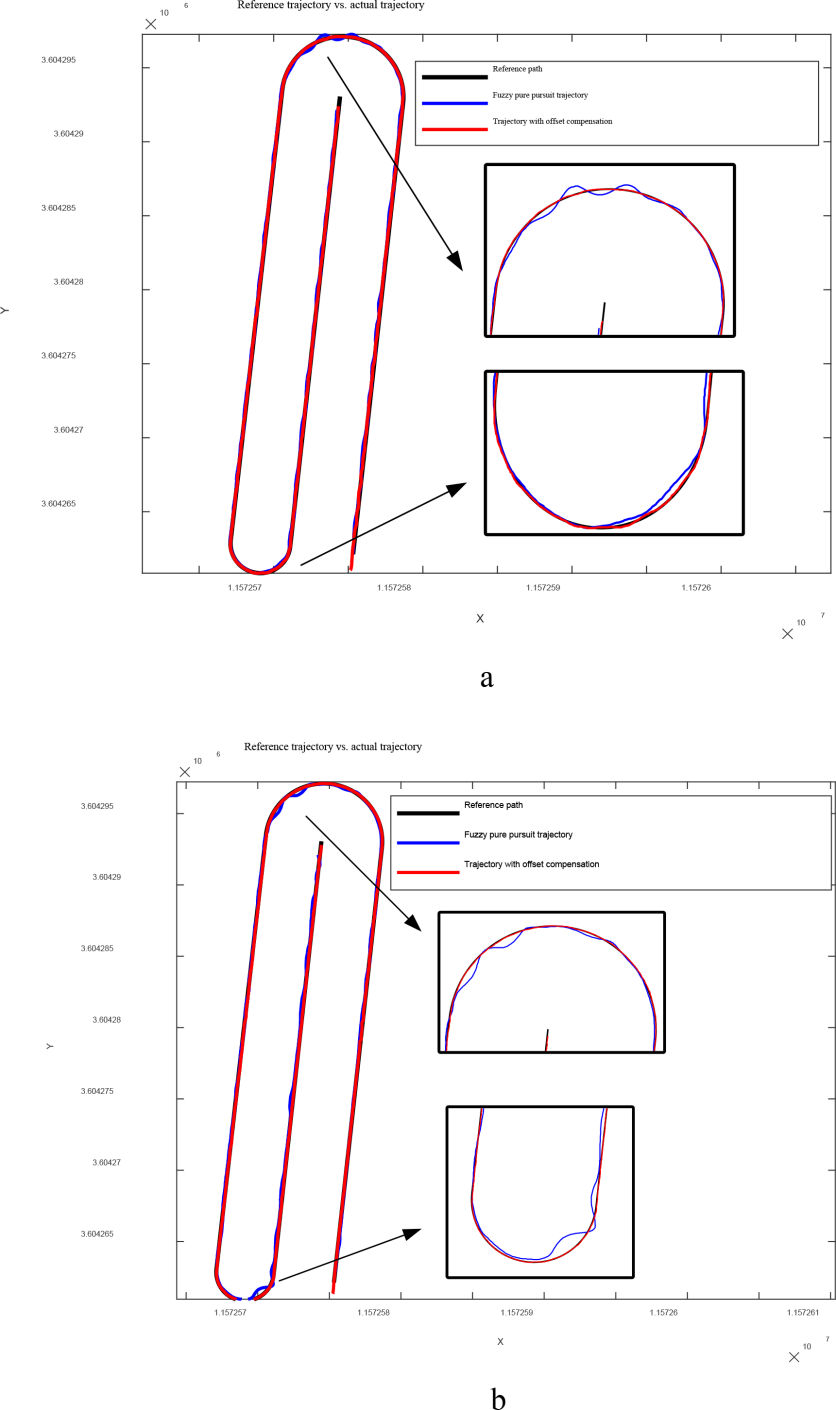
Path-tracking comparison at different speeds. **(a)** Set vehicle speed v=0.35m/s, **(b)** Set vehicle speed v=0.75m/s.

**Figure 14:**
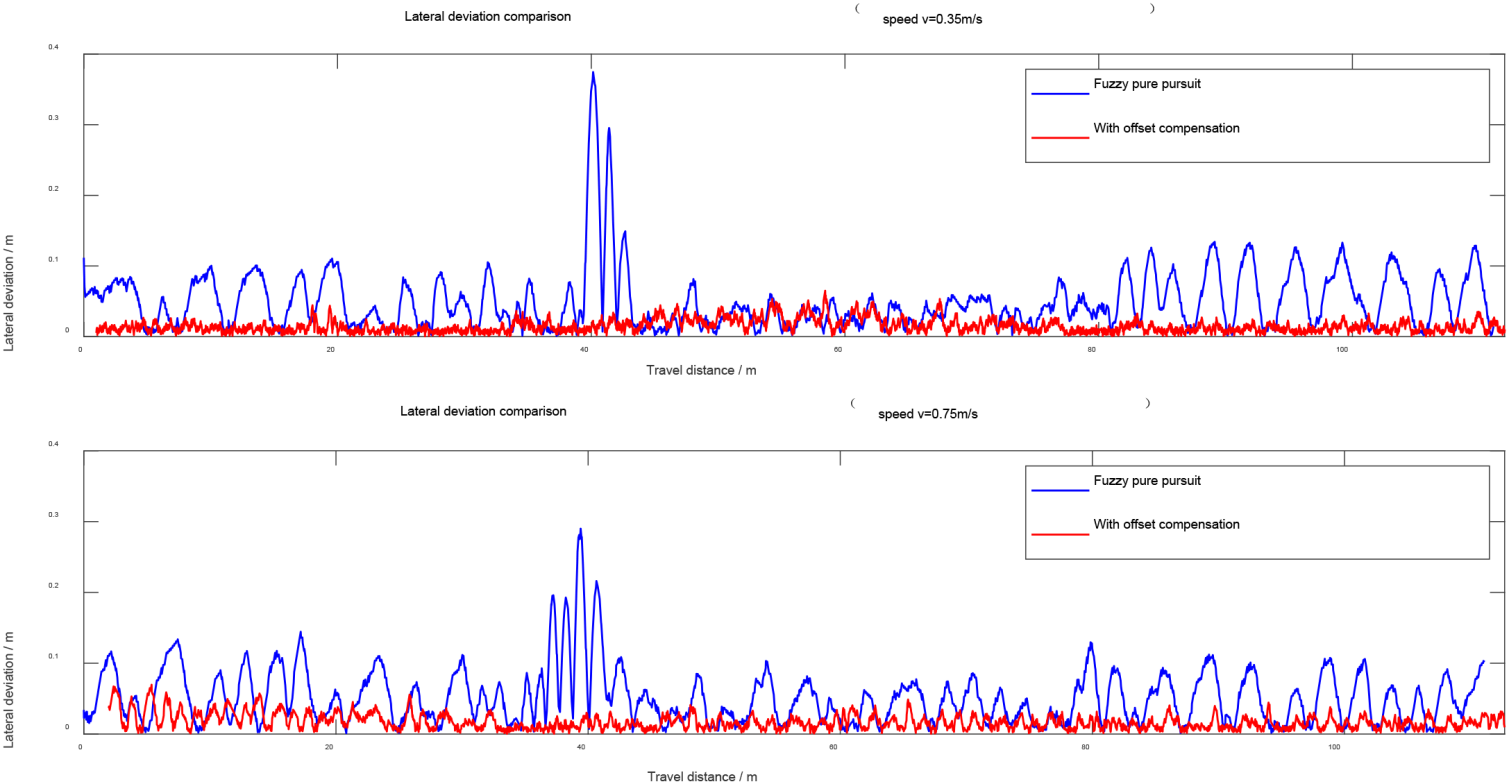
Lateral deviation comparison curves under different speed conditions.

The vehicle was tested under two control modes: “fuzzy Pure Pursuit” and the proposed “slip-aware look-ahead point offset” strategy. The test speeds were set to 0.35 m/s and 0.75 m/s. For each mode and speed, the vehicle trajectory and tracking deviations were recorded. The tracking performance results are shown in **Figure 13** and **Figure 14**.

To ensure data reliability, each experiment was repeated three times. The statistical results of the repeated trials are summarized in **Table 4**, where the plotted results correspond to the A1 and B1 test groups.

**Table 4:** Path-tracking experimental data.

Speed/ ( $m\cdot s^{-1}$)	Test group		Maximum lateral deviation/m	Average lateral deviation/mo	Standard deviation
0.35	A1	A1-m	0.375	0.083	0.045
		A1-n	0.082	0.041	0.043
	A2	A2-m	0.341	0.079	0.052
		A2-n	0.097	0.041	0.047
	A3	A3-m	0.632	0.085	0.049
		A3-n	0.088	0.039	0.044
0.75	B1	B1-m	0.293	0.125	0.048
		B1-n	0.108	0.053	0.047
	B2	B2-m	0.318	0.087	0.051
		B2-n	0.112	0.049	0.046
	B3	B3-m	0.335	0.091	0.050
		B3-n	0.106	0.052	0.045

In the experiment groups, *m* denotes the fuzzy path tracking algorithm, while *n* denotes the path tracking algorithm with the proposed offset compensation strategy.

From the results shown in the figures, the traditional fuzzy Pure Pursuit method exhibits varying degrees of deviation in both straight and turning segments, with larger deviations occurring at higher vehicle speeds. The deviation becomes particularly pronounced in the turning regions at both ends of the path, where differential slip between the tracks leads to significant trajectory drift.

In addition to the trajectory comparison, the internal responses of the proposed control framework were examined to verify the effectiveness of each module. The EKF-estimated slip ratios exhibit clear consistency with the expected soil-condition settings and operational behaviors. Specifically, the left track shows a higher estimated slip level during left-turn segments, while the right track presents a larger slip ratio in right-turn segments, matching the physical characteristics of differential-steering tracked vehicles. Although the true slip ground-truth is not directly measurable in field conditions, the estimated slip presents smooth temporal evolution without abrupt jumps, indicating stable EKF convergence.

The velocity commands generated by the controller are also smooth due to the low-pass filtering, and no oscillation or overshoot is observed throughout the experiments. During curved segments, the inner track velocity decreases while the outer track velocity increases, which aligns with the vehicle’s turning geometry. These observations confirm that the slip-aware offset mechanism and the adaptive tuning modules operate correctly and contribute jointly to the improved tracking performance.

In contrast, after applying the slip-aware offset compensation strategy, the vehicle trajectory aligns much more closely with the reference path. The straight-line segments display improved stability, and the turning deviations are effectively corrected. Overall, the proposed method demonstrates higher tracking accuracy and smoother trajectory performance.

According to **Table 4**, when the vehicle speed is 0.35 m/s, both the maximum and average lateral deviations are significantly reduced after applying the offset compensation strategy. The maximum lateral deviation is reduced by 78.1%, and the average lateral deviation decreases by 50.6%.

When the speed is increased to 0.75 m/s, the maximum lateral deviation is reduced by 63.1%, while the average lateral deviation decreases by 57.6%.

These results indicate that the proposed slip-aware offset compensation control method effectively suppresses lateral deviation at different operating speeds, ensuring stable and robust path tracking performance.

## 5 Conclusions and future work

1. To address the degradation of path tracking accuracy caused by slip in tracked agricultural machinery operating in complex farmland environments, this study proposes a slip-aware look-ahead point offset path tracking control method. Building upon the traditional Pure Pursuit algorithm, the method incorporates a fuzzy controller for adaptive optimization of the look-ahead distance, employs an extended Kalman filter to achieve online slip-ratio estimation, and integrates an improved particle swarm optimization algorithm to determine the optimal target-point offset angle. These enhancements significantly improve the adaptability and robustness of the controller under slip conditions.

2. Both simulation and field experiments demonstrate strong tracking performance of the proposed method. At operating speeds of 0.35 m/s and 0.75 m/s, the maximum lateral deviation is reduced by 78.1% and 63.1%, respectively, while the average lateral deviation is reduced by 50.6% and 57.6% compared with the fuzzy control baseline. These results verify the practicality and engineering feasibility of the proposed approach, showing its capability to meet operation requirements in complex farmland environments.

3. Future work will further incorporate vehicle dynamics and operation loads, and include more comprehensive benchmarking against classical dynamic controllers such as LQR and MPC by embedding the proposed slip-aware offset mechanism into these frameworks. In addition, lightweight optimization algorithms will be explored to enhance real-time performance, and multi-machine collaboration and intelligent scheduling platforms will be integrated to extend the applicability of the proposed method to large-scale intelligent agricultural machinery systems.

## Data Availability

The original contributions presented in the study are included in the article/supplementary material. Further inquiries can be directed to the corresponding author.
